# A Mixed Methods Study Examining Citizenship Among Youth With Mental Health Challenges

**DOI:** 10.3389/fpsyt.2022.852947

**Published:** 2022-05-18

**Authors:** Gerald Jordan, Laura Burke, Julia Bailey, Sof Kreidstein, Myera Iftikhar, Lauren Plamondon, Courtney Young, Larry Davidson, Michael Rowe, Chyrell Bellamy, Amal Abdel-Baki, Srividya N. Iyer

**Affiliations:** ^1^Department of Psychiatry, McGill University, Montreal, QC, Canada; ^2^Yale Program for Recovery and Community Health, Department of Psychiatry, Yale University, New Haven, CT, United States; ^3^Department of Psychiatry, Université de Montréal, Montreal, QC, Canada

**Keywords:** citizenship, community, youth, mental health, intersectionality, mixed methods

## Abstract

**Introduction:**

Multiple stakeholders have recently called for greater research on the barriers to citizenship and community belonging faced by people with mental health challenges. Citizenship has been defined as a person’s access to the rights, roles, responsibilities, resources and relationships that help people feel a sense of belonging. Factors that may impact citizenship include financial precarity; intersecting forms of marginalization and oppression (e.g., racism); and the mental health care people receive. Research has yet to examine experiences of citizenship among youth with mental health challenges. To address this gap, this study will examine how youth experience citizenship; predictors of citizenship; how citizenship shapes recovery; and the degree to which youth are receiving citizenship-oriented care.

**Methods:**

The research objectives will be evaluated using a multiphase mixed methods research design. Quantitative data will be collected cross-sectionally using validated self-report questionnaires. Qualitative data will be collected using a hermeneutic phenomenological method using semi-structured interviews and focus groups.

**Analyses:**

Multiple stepwise regression analyses will be used to determine predictors of citizenship and if of citizenship predict recovery. Pearson correlations will be computed to determine the relationship between participants’ perceived desire for, and receipt of citizenship-oriented care. Phenomenological analysis will be used to analyze qualitative data. Findings will then be mixed using a weaving method in the final paper discussion section.

**Conclusion:**

Findings from this study may support the development of citizenship-oriented healthcare in Canada.

## Introduction

Mental health challenges are the difficulties people may experience with emotions, thoughts, behaviors, and functioning; and may reflect symptoms, difficult experiences or diagnoses (such as schizophrenia). Many activists and scholars have described how neoliberal politics, economics, institutions, and social policies can lead to mental health challenges. These policies have sought to reduce market restrictions, weaken labor unions, privatize or reduce funding to provincial and federal programs, and promote free trade across national and provincial borders (1). The impacts of such policies, systems, and structures include feeling socially disconnected from, and more competitive with others (2, 3); precarious or overemployment (4, 5); poverty (6); rising personal debt (7); and homelessness (8). Neoliberalism and mental health challenges often intersect with experiences of stigma, colonialism, racism, heterosexism, and gender-based discrimination to further alienate young people who struggle with their mental health (9–12). Together, these experiences may disrupt young people’s sense of citizenship and belonging within their communities.

The 5 R’s framework is the most well-established model of citizenship as it pertains to people with mental health challenges and is based on work with adults living in the United States (13). The model defines citizenship as the strength of a person’s access to the rights (e.g., freedom to associate), responsibilities (i.e., actions that are important to people), roles (i.e., positions occupied in society), and resources (e.g., money) needed to live a full and meaningful life; as well as their relationships (e.g., support networks, close ties, and community connections) and socially validated sense of belonging. Full citizenship is contingent upon people with mental health challenges both participating in social and community life, as well as being valued for their participation by others in their community (13). This model highlights the need for both instrumental and affective elements of citizenship (14); and supports the inclusion and participation of persons with mental health challenges within their communities (15).

Barriers and facilitators of citizenship among adults with mental health challenges have been examined in four qualitative and one quantitative study. Barriers to citizenship include having limited choices; experiencing poverty; experiencing internalized and structural stigma, sanism and other forms of discrimination; feeling unsafe in one’s community; and not having legal citizenship (16–18). In contrast, facilitators of citizenship include following the hidden scripts and rules of one’s community; not having a criminal record and/or experience of oppression under carceral law; being satisfied with social relations and health; and feeling a sense of community social connectedness (19).

Recovering from a mental health challenge involves finding ways to live a full, meaningful life despite the difficulties associated with such experiences. Recovery is a personal and non-linear process that may involve finding ways to manage symptoms, overcome external and internalized sanism, achieve a positive sense of self, find meaning in life (20) and build self-compassion. Researchers, policy makers, mental health care practitioners, activists, and community members have recently called for greater attention to the underlying material, social, cultural and political realities that influence recovery from mental health challenges and struggles. Given that one’s sense of citizenship is underpinned by such factors, experiencing a lack of, as well as barriers to citizenship may in turn affect if and how people recover from mental health challenges (21). In support of this, studies have shown moderate correlations between measures of citizenship and recovery (22); that citizenship-based interventions have led to decreased substance use (23); higher levels of satisfaction with social activities, finances and work; improvements in overall quality of life (24); and feeling like people’s place in their communities has been reclaimed (14, 25, 26).

This body of work may help people with mental health challenges reclaim the material, social and political resources needed to achieve full recovery (21). However, aspects of this work may not be entirely applicable to young people with mental health challenges living in Canada. The 5 Rs model of citizenship is based on work with adults living in the United States, which has its own social and political realities that may make the model not entirely applicable to young people living in Canada. In addition, it is unclear if the current mental health supports youth receive address elements of citizenship-oriented care, or if youth feel these supports and resources (often existing within institutional settings) would aid in their healing.

Youth between the ages of 14 and 25 with and without mental health challenges in Canada face developmental, generational and political circumstances that may impact their mental health and their sense of citizenship. As all young people develop and mature, they develop a sense of identity in relation to their social context (27). They begin to foster an understanding of their place in the world and in their communities, and such understandings may be influenced by varying factors (e.g., racism, poverty, urbanicity) (28). A Statistics Canada report on the status of Canadian youth highlighted that in recent years, youth in general are more likely to openly and/or personally identify with marginalized identities, including, but not limited to LGBTQ+ identities, than older generations (29). Compared to the total population, people who identify as LGBTQ+, female, disabled and racialized experienced higher levels of discrimination. In addition, religious minorities also face high levels of discrimination in Canada (30). Such experiences of discrimination and oppression may reduce feelings of citizenship and alienate youth with mental health challenges from their communities, in turn making recovery from such challenges more difficult.

Also highlighted in the report is that young people are more likely to be digitally connected to others relative to older generations; however, disparities in access to high-speed internet, especially among youth living in rural locations (31), remain present. Youth living in poverty or in unhoused conditions may also face disparities with respect to digital access (32). This issue is of particular importance in light of the COVID-19 pandemic, during which much of life in Canada has moved online, and community for many youth may be restricted to digital spaces. Youth with mental health challenges experiencing such inequities may feel alienated from digital and offline communities, thereby impacting their sense of citizenship.

The report also notes that the cost of post-secondary education has risen past the rate of inflation, leaving younger Canadians with higher levels of debt and financial stress, relative to older generations. A separate report has revealed that younger Canadians increasingly cannot afford to rent or purchase a home, which may prevent young people from feeling like they have security and a place within their communities (33). Such constraints to young people’s financial resources may limit the extent to which they can participate in society, thereby impacting their sense of citizenship. Together, these disparities, which are particularly salient among youth, may influence their citizenship, and in turn their recovery from mental health struggles.

The quality of care that people receive has a robust impact on people’s recovery and senses of community (34); and it is thus important to evaluate the perceived recovery orientation of such services. While young people receive care in varying settings and contexts, recent investments at the Canadian national and provincial levels have resulted in the development of integrated youth mental health services. These services are often located within communities and serve as a “one-stop-shop” where youth can address various psychological and social needs. ACCESS Open Minds/Esprits ouvertes was the first network of such services established in Canada, and its aims are to reduce unmet mental health needs; ensure rapid access to care that is available within 30 days; engage young people and families in care; and eliminate transitions between child and adult services by allowing continuity of care for youth between 11 to 25 years of age. It is thus important to evaluate how such care influences citizenship among young people (35, 36). Other similar services have been established throughout Canada in the province of Quebec (Aires ouverts), Ontario (Youth wellness hubs) and British Columbia (Foundry BC) and operate under similar principals.

Finally, given the importance of feeling like a citizen within one’s community (20), and the growing role of integrated youth mental health services in Canada, it is important to evaluate the extent to which such services support young people’s senses of citizenship. It is also important to evaluate such support against young people’s needs for citizenship-oriented care to be delivered within such services as proof of concept for future citizenship-oriented interventions.

Given the nuances that young people living in Canada face as well as the lack of qualitative and quantitative research in this area, there is a strong need for research using mixed methods to understand how youth experience citizenship; barriers to citizenship; and the relationship between citizenship and recovery. There is also a need to unpack the desirability for citizenship-based Canada. There is a need for qualitative research to unpack the subjective experiences of citizenship, as well as quantitative research demonstrating predictive relationships between citizenship, barriers to citizenship, and recovery.

### Research Objectives

To address these knowledge gaps, this study will employ mixed methods to evaluate several objectives among youth between the ages of 14 and 25 with mental health challenges and living in Canada. The **qualitative research objectives** are to explore (1) how youth experience a sense of citizenship, (2) barriers to citizenship, (3) and how one’s sense of citizenship influences their experience of recovery. The **quantitative objectives** will be to examine (1) predictors of citizenship, (2) to determine if citizenship predicts recovery, and (3) to compare current and desired levels of citizenship-based care.

## Methods

### Paradigm

The project will use different methods (i.e., quantitative and qualitative) and is being conducted by a multidisciplinary team of people with varying types of expertise, professional roles, and experiences of marginalization and oppression. Dialectical pluralism will therefore be the metaparadigm guiding this study, which offers the intellectual framework for incorporating different standpoints, philosophical positions and methods within one overall study (37). Specifically, a post-positivist paradigm will guide the quantitative component (which acknowledges the existence of a measurable objective reality that is nonetheless influenced by subjective perceptions) while a constructivist/interpretivist paradigm will guide the qualitative component (which acknowledges the existence of multiple truths and interpretations of reality that are based within social, political, economic and historical contexts).

### Theoretical Framework

This study will be guided by the theoretical framework of intersectionality (38), which will enable us to examine how multiple aspects of a person’s identity interact in complex ways to influence young people’s sense of citizenship. For instance, we may examine how a person’s gender and religious faith shape people’s sense of citizenship in the context of recent laws barring religious symbols being displayed in certain work educational settings (39) in the province of Quebec.

The three factors of intersectionality applied in this study are to place (a) marginalized people as the starting point of analysis (i.e., young people with mental health challenges); (b) to examine how the impact of multiple other identities (e.g., race, class, gender, etc.) intersect to produce health outcomes and (c) explore how these intersections are experienced alongside forms of systemic, interpersonal, and internalized oppression (e.g., racism, classism, sexism, transphobia, etc.) (40). In addition, we will examine how the qualitative findings can be traced back to economic, political and social policies (2).

### Setting and Participants

Participants will be recruited online through ACCESS Open Minds/Esprits ouverts, a pan-Canadian youth mental health network of 14 services providing care for youth aged 14–25. We will distribute our recruitment poster via an electronic listserv within this organization, and youth who are interested in participating will be invited to click on a link to complete the surveys or interview. We will also share our poster on the network’s twitter account. If our recruitment numbers fall short, we may seek to recruit participants through other means (such as physical or online posters in other mental health service centers serving youth).

People will be eligible to participate in the quantitative components of the study if they are between the ages of 14 and 25; can communicate in and understand English or French; are currently receiving services at an integrated youth mental health service in Canada; and self-report having experienced a mental health challenge. People will be eligible to participate in the qualitative component of the study if they meet these criteria as well as feel excluded or like they do not have all the privileges of citizenship; and have felt discriminated against and have experienced oppression.

### Overall Study Design

**FIGURE 1:**
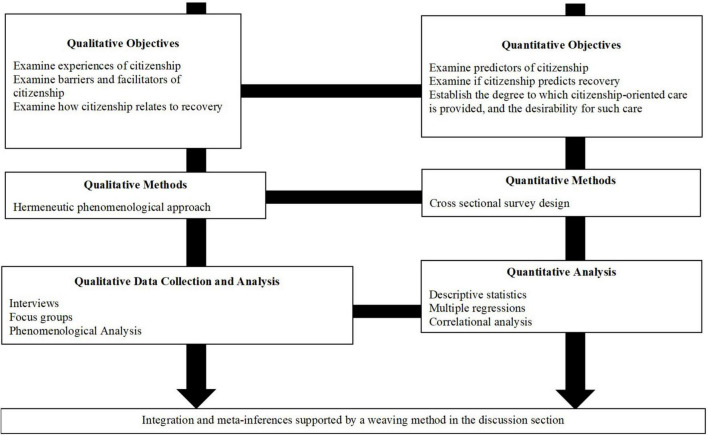
Overall methodological approach for the study.

The study objectives will be evaluated using a multistage mixed methods research design whereby qualitative and quantitative components will be conducted over the course of several years and integrated at various junctures. This design is appropriate for multi-year projects requiring several stages and methods to inform a larger program of research (Figure 1) (41). Specifically, one qualitative component and two quantitative components will be conducted to address the objectives. Participants will not be required to participate in all components of the study; if they wish, they can only complete one, two or three components.

Using mixed methods will allow us to rely on the strengths inherent in different approaches; and will help us thoroughly address the full range of knowledge gaps related to experiences of citizenship among young people with mental health challenges living in Canada.

Given the limited knowledge the topic, priority will be given to the qualitative component of this study. All components will be conducted concurrently.

This study has received Ethical Approval from the Douglas Hospital Research Centre Ethics Board. Informed consent will be obtained from all participants prior to participation. To consent, participants themselves will be given the opportunity to click on a button stating “I consent to participate in this study” on an online consent form prior to beginning the study.

#### Qualitative Methods

The qualitative component of this study will be used to evaluate how young people with mental health challenges experience citizenship; barriers and facilitators of citizenship; and how their sense of citizenship shapes their recovery.

A hermeneutic phenomenological method will guide the qualitative evaluation of this component. The aim of hermeneutic phenomenology is to help uncover how human experience is lived and situated within the world itself; and is a method that fits well within various types of mixed methods approaches (42). This approach recognizes that research participants and researchers are self-interpreting beings that exist within a social world, history, and context (43). In keeping with our theoretical framework, we will seek an understanding of how neoliberalism and the various intersections of participants’ identities interact to shape the topics being explored throughout the qualitative component.

We will conduct semi-structured, individual online interviews to uncover participants’ experiences of citizenship; facilitators of citizenship; and how citizenship shapes experiences of recovery and healing. First, participants will answer demographic questions using an online survey platform (which will take approximately 5-10 min to complete). Three questions will be asked during the interviews, which will allow the interviews to remain open to what participants decide is important to discuss regarding the topic. Interviews are expected to last between 45 min and 1 h and will be audio-recorded and be conducted by the first author. Detailed notes and analytical memos will be kept throughout the study to enhance rigor. Interviews will be audio-recorded and transcribed verbatim. Once the transcriptions have been checked for accuracy, the recordings will be deleted. Each word and vocalization will be transcribed. To maintain rigor, researchers on this project will be reflexive and examine the impact of how their academic privilege and “multiple brought selves” may shape the interpretation of the findings (e.g., their brought selves, situational selves, and research-based selves) (44). Interviews will take place in English and French. Participants will receive a gift card totaling up to $50.00 for completing the interview.

All people who participated in the interviews will then be invited to two separate, semi-structured focus group where the themes will be presented to ensure that they adequately represent the common story across interviews. One focus group will be conducted in English while the other will be conducted in French. The focus group will take place over Zoom and last between 45–60 min. Since we are only interested in ascertaining if our analysis captured participants’ collective experiences and in refining our themes, focus groups will not be recorded. Only a researcher’s handwritten notes of the event will be kept. Modifications to the themes will be made based on the focus group feedback. Participants will receive a $40.00 gift card for their participation in the focus group. The focus groups will take place in English and French.

#### Qualitative Sampling Strategy

We will employ a purposeful sampling technique to recruit approximately 10-15 participants with mental health challenges for interviews. This recruitment target was chosen based on the availability of funds for this project. Specifically, we will seek to recruit people who feel or have felt excluded and discriminated against and/or do not have a sense of citizenship. All participants will be invited to participate in the focus group with other interviewees to unpack the study findings on a later date.

#### Qualitative Materials

In keeping with hermeneutic phenomenology and our theoretical framework, three questions will be asked during the *interviews*: (1) What does “being a citizen” mean to you? (2) What makes feeling like a citizen challenging or easy? and (3) How has your sense of citizenship influenced your recovery from your mental health challenge (s)? Our guide also contains probes assessing experiences of discrimination, financial stress, access to digital technology, and the quality of mental healthcare received. Our interview will remain flexible to discussing what participants themselves may feel is important to about the topic outside of what is contained in the guide. All interview data (stemming from the guided questions to unguided questions) will be analyzed and included in our final report. During *focus groups* participants will be asked: (1) Is what we just presented consistent with your experience of citizenship? (2) Did what we present make sense to you? (3) Is there anything that we should reconsider or think differently about related to what we presented?

#### Quantitative Methods

We will employ a cross-sectional survey design to examine current and desired levels of citizenship-oriented care; predictors of citizenship and the degree to which a sense of citizenship predicts recovery.

To measure current and desired levels of citizenship-oriented care, participants will complete questionnaires assessing demographic and identity-based characteristics; the extent that their current mental healthcare providers have addressed citizenship (focusing on 12 core aspects of citizenship-oriented care); and the extent to which participants desire their mental healthcare providers address these 12 aspects (16). Participants will be given the choice to enter their email address in a raffle for a $50.00 gift card for completing this component. Pilot data suggest that approximately 5–10 min are expected to be required to complete these questionnaires.

To assess the other objectives outlined above, participants will be asked to complete self-report measures of demographic and identity-related characteristics; their senses of citizenship and recovery; experiences around stigma and other forms of discrimination; access to digital technology; the recovery orientation of their mental health services; as well as their financial status. Participants will be given the choice to enter their email address in a raffle for a $100.00 gift card for completing this component. Pilot data suggest that approximately 20–45 min are expected to be required to complete these questionnaires.

#### Quantitative Sampling Strategy

A purposeful sampling strategy will be employed to oversample participants from marginalized demographics. This sampling methodology is in line with recommendations for thorough and ethical best practices for examining intersectionality within quantitative studies and will help us recruit participants who are typically underrepresented in research (45).

270 participants will be recruited to evaluate predictors of citizenship and to determine if citizenship predicts recovery. 100 participants will be recruited to determine current and desired levels of citizenship-based care.

To examine rates and predictors of citizenship, as well as the degree to which citizenship predicts healing and/or recovery, we will aim to recruit participants of varying marginalized ethnicities, genders, sexualities, and religious (54 participants per identity category). We will arrive at a total sample size of approximately 270; which is, according to a power analysis conducted in G*Power (version 3.1), the number of participants needed given 24 predictor variables, an 80% power level and moderate projected effect size.

Once we have recruited 135 participants, we will perform an interim analysis to determine if we are meeting recruitment targets for each group. If not, we will purposefully sample members of under-recruited identities and demographics. For example, if we only recruited 5 participants who are not heterosexual, we will adjust our recruitment strategies and poster to explicitly state that we are particularly seeking participants from these groups.

The same sampling strategy will be employed to examine participants’ current and desired levels of citizenship-oriented care. However, we will aim to recruit 20 participants from each of the aforementioned groups.

#### Quantitative Measures and Materials

All measures will be available in English or French, and participants will be able to complete questionnaires in either language (Table 1). Questionnaires without French versions have been translated by a professional translator into French in line with the World Health Organization’s instructions for translation and adaptation of instruments (46). Questionnaires will take approximately 45 min to complete.

**TABLE 1:** Details of included measures.

Name of questionnaire	Domains	Example items
Brief Citizenship Outcome Measure (16)	Responsibilities toward others; knowledge about community; respect for personal decisions; access to employment; connection with others; being part of something bigger than oneself; freedom to worship; right to protect oneself; right to second chances; caregiving; discrimination; safety.	“How much do you feel your current mental health services and/or clinicians have/should collaborated with you to fight discrimination?”
Citizenship Outcome Measure (47, 48)	Personal responsibility; government and infrastructure; caring for self and others; civil rights; legal rights; choices; world stewardship.	“You do things to take care of your home; you have access to services at a bank; other people depend on you; you have equal opportunities; there are laws that will protect you; your personal decisions and choices are respected.”
Everyday Discrimination Scale (49)	Overall discrimination.	“You are threatened or harassed; what are the main and other reasons for these experiences (e.g., ancestry or national origin, gender, race, skin color, age, religion, height, weight, sexual orientation, education or income level, physical disability, physical appearance, band or tribe)?”
Adolescent Discrimination Distress Index (51)	Educational discrimination; institutional discrimination; peer discrimination.	“You were given a lower grade than you deserved; you were hassled by police; you were called racially insulting names.”
Financial Stress Scale for Undergraduate Students (52)	Financial situation; debt, credit and loans; expenses.	“Living paycheque to paycheque; having a low credit score; paying taxes.”
Gender Minority Stress and Resilience Measure (53)	Gender-related discrimination; rejection; victimization.	“I have experienced difficulty getting identity documents that match my gender identity; I have been rejected at school or work because of my gender identity or expression; I have had my personal property damaged because of my gender identity or expression.”
Minority Stress Scale (54)	Structural stigma; enacted stigma; expectations of discrimination; discrimination from family members.	“I won’t be able to adopt children because of my sexual orientation; I have experienced physical aggression because of my sexual orientation; I feel excluded from my society because of my sexual orientation; I think my family would not accept me because of my sexual orientation.”
Religious Discrimination Scale (55)	Perceived prejudice; closet symptoms; negative labels.	“I was passed over for opportunities due to my religion; I felt inclined to keep my religious affiliation private; I have heard people make unfriendly remarks about my religion.”
The Stigma Scale (56)	Discrimination; disclosure; positive aspects of mental illness.	“I have been discriminated against in education because of my mental health challenges; I worry about telling people I receive psychological treatment; having had mental health challenges as made me a more understanding person.”
The Australian Centre for Sustainable Business and Development (57)	N/A	“Internet upload speed is appropriate for my household; the cost of the internet connection is reasonable for my household; learning to use the internet is easy; I feel positive toward the use of the internet.”
Recovery Assessment Scale (58)	Personal confidence and hope; willingness to ask for help; goal and success orientation; reliance on others; no domination by symptoms.	“I can handle what happens in my life; I ask for help when I need it; I have goals in life that I want to reach; even when I don’t care about myself, other people do; my symptoms interfere less and less with my life.”
Recovery Self-Assessment Revised (59)	Life goals; diversity of treatment options; choice; individually tailored services; inviting space.	“Staff help me to develop and plan for life goals beyond managing symptoms or staying stable; I am given opportunities to discuss my sexual needs and interests when I wish; I can change my clinician or case manager if I want to; this program offers specific services that fit my unique culture and life experiences; staff welcome me and help me feel comfortable in this program.”

A demographic questionnaire will be used to measure characteristics pertaining to participants’ sex, genders, sexual orientation, religious affiliation (and if they wear religious symbols); education and employment status; country of birth; relationship statuse; ethnicities and races; Indigenous identities, background, affiliation, and band membership; as well as the people they are domiciled with. This questionnaire will also assess participants’ emotional, psychological or mental health conditions, symptoms and experiences; as well as the degree to which participants feel limited and/or affected by these conditions, symptoms, and experiences. The questionnaire will assess whether participants have any additional health conditions, symptoms, and/or disabilities. Finally, this questionnaire will ask about how participants define the word “community.”

Participants’ perceived levels of current and desired citizenship-oriented care will be measured using an adapted version of the *Brief Citizenship Outcome Measure*. This questionnaire contains 12 items that assess core aspects of citizenship that were identified in a previous study (16). To measure current levels of citizenship-oriented care, participants will be asked, “How much do you feel your current mental health services and/or clinicians have collaborated with you to” and to rate the degree to which such services and professionals supported each of the 12 aspects. They will also be asked where they receive mental health services. To measure the degree to which participants desire citizenship-oriented care, participants will be asked about the extent to which mental health services and professionals should support each of the 12 core aspects. These measures were created by the study team; therefore, no psychometric properties for these measures are available.

Young people’s sense of citizenship will be measured using the *Citizenship Outcome Measure* (47, 48). The scale contains seven domains rated with 46 items on a 5-point Likert scale (not at all/never – a lot/very often). The scale has acceptable internal consistency (α = 0.96) and test-retest reliability (*r* = 0.66); high convergent and discriminant validity with related concepts (e.g., sense of community, social capital, etc.). The scale has been translated into French and has similar psychometric properties (21).

We will measure overall experiences of discrimination using the original version of *the Everyday Discrimination Scale* (49). This widely used questionnaire includes nine items (e.g., you are threatened or harassed) measured on a six-point Likert-type scale (never -almost everyday) that map onto one overall factor. The scale has excellent convergent validity with constructs such as psychological distress, depression and anxiety; high internal consistency (α = 0.91) and test-retest reliability (α = 0.74) (50).

Racial and ethnic discrimination will be measured using the *Adolescent Discrimination Distress Index*. The questionnaire contains 15 items, to which participants are to indicate if they ever experienced each form of discrimination (no/yes), and the degree to which each form of discrimination was upsetting (e.g., not at all – extremely). The measure has been developed for use with adolescents, and measures racial and ethnic discrimination in three domains mapping onto one factor. The scale has acceptable inter-item (α > 60) and test-retest (*r* = 0.53) reliability (51).

Financial stress will be measured using the *Financial Stress Scale for Undergraduate Students* (52). The questionnaire assesses how often participants thought about 13 sources of financial stress on a four-point scale (never – all the time). The overall scale has high internal consistency (α = 0.87), high convergent validity with measures of everyday stress; and acceptable factor loadings, with eigenvalues ranging from 0.58 to 0.85.

Data on gender discrimination among transgender and gender-non-conforming people will be measured using the *Gender Minority Stress and Resilience Measure* (53). In keeping with our intent on exploring the impact of oppression and discrimination on citizenship, three subscales will be included. Participants will be asked to indicate if they have experienced these forms of discrimination according to the following response options: never, yes before the age of 18, yes after the age of 18, and yes in the past year. The scale is strongly correlated with mental health outcomes such as depression, suggesting it has strong criterion and convergent validity. Items of the scale have acceptable factor loadings, with eigenvalues ranging from 0.37 to 1.00.

Sexuality-based discrimination data will be using the *Minority Stress Scale*. The scale measures such discrimination using 21 items ranked on a five-point scale (e.g., completely disagree – completely agree) and also has a “not applicable” option. Items of the scale have acceptable factor loadings, with eigenvalues above 0.40, and the scale has strong convergent validity as evidenced by significant correlations with measures of perceived stress (54).

Religious discrimination data will be measured using the *Religious Discrimination Scale* (55). This questionnaire measures religious discrimination using 11 items that are rated on a five-point scale (never – always). The scale was revised by the study team to also include a “not applicable” option so that people who are not religious or spiritual could complete the scale. Items of the scale have acceptable factor loadings, with eigenvalues above 0.64. In addition, reliability scores are adequate for the overall scale (α = 0.80) as well as its subscales (α = 0.78–0.89).

Mental health stigma will be measured using the *Stigma Scale*, which will measure experiences of stigma across 28 items rated on a five-point scale (strongly disagree – strongly agree) (56). The scale has a high affirmation of items, high test-retest reliability (κ > 0.71), high internal consistency (α = 0.87), and strong concurrent validity.

Experiences relating to digital access will be assessed using a subscale of a questionnaire developed by the *Australian Centre for Sustainable Business and Development* (57) which measures perceived accessibility, usefulness, affordability, efficacy and ease of use of the internet. The subscale contains 15 items that are ranked on a five-point scale (strongly agree – strongly disagree) and contains a “not applicable” response choice for each item. The scale was developed in English and the psychometric properties of the scale have not been released. We will evaluate the psychometric properties of the scale before including it in our analyses. This scale was also translated into French by the study team and has not yet undergone psychometric evaluation.

Recovery will be measured using a brief version of the *Recovery Assessment Scale*. The questionnaire measures aspects of recovery using 24 items that are rated on a five-point scale (Strongly Disagree – Strongly Agree). The 24 items map onto five domains Both English and French versions have the same factor structure, and acceptable internal consistency coefficients (α = 0.76 – α = 0.97). The English version has high test-retest reliability, ranging from 0.65 to 0.88. The scale has strong convergent validity, as demonstrated by its strong correlations with constructs such as psychological well being, social functioning and participation (58).

The recovery orientation of services will be measured using the Person-in-Recovery version of the *Recovery Self-Assessment – Revised* (59). This questionnaire contains 32 items that measure the degree to which the mental health services that persons receive care from have incorporated principles of recovery-oriented care across six domains on a five-point scale (strongly disagree – strongly agree). The psychometric properties of the English version suggest the scale has high internal consistency (α = 0.96) and test-retest reliability (*r* = 0.84); as well as strong convergent validity, as suggested by correlations between the scale scores and constructs such as personal optimism. The psychometric properties of the French version of the scale have not been published.

## Analysis

### Qualitative Analysis

Transcripts will be analyzed using the phenomenological approach developed by a member of the study team (60). Specifically, entire transcripts will be condensed into one-page summaries covering the key points described by participants using their own words, with the aim of uncovering the underlying story behind participants’ experiences. The summaries will be extracted into a narrative format that will be summarized in the first-person. This method will allow us to eventually move beyond participants’ individual experiences to uncovering the shared meaning structure within all participants’ narrative accounts. Given our aim of understanding participants’ narratives, this method is more suitable than methods which rely on line-by-line coding and the formation of common patterns or themes among participants, such as thematic analysis. To be consistent with our theoretical framework, we will intentionally examine how intersecting forms of participants’ identities impact participants’ experiences. Once the transcripts are summarized, the research team will identify the shared meanings and themes within them. The research team will then convene to discuss and refine each theme. Later, themes generated through the analysis will be presented during focus groups and participants will be asked to refine or make suggestions to the themes. The first author has extensive experience conducting interviews and focus groups and will facilitate each group.

### Quantitative Analyses

Predictors of citizenship will be determined using a stepwise multiple regression analysis. Terms reflecting identity will be entered into the first block followed by the main predictor variables. In line with current recommendations for testing the impact of intersectionality on outcomes in quantitative research (61, 62), we will build interaction terms reflecting components of participants’ identity. We will base our interaction terms on the qualitative findings. If interaction terms cannot be built because of power considerations, we will employ an “additive” strategy, whereby we will add ethnicity, race, gender, sexuality, and religion separately to determine both their independent (within single regression blocks) and additive (together within one regression block) impact on the outcome measures. We will test for multicollinearity among the main predictor variables through examining Pearson correlations between each variable. We will also determine if differences in French and English questionnaire responses exist and will control for language if differences are detectable.

A multiple stepwise regression will be conducted to determine if overall scores on the Citizenship Outcome Measure predict recovery.

Pearson correlations will be computed between the extent to which participants perceive that their mental healthcare providers address citizenship and the extent to which participants feel their providers should be addressing citizenship. The internal consistency and factor structure of the adapted version of the Brief Citizenship Outcome Measure will be evaluated (Table 2).

**TABLE 2:** Quantitative analyses 1.

Quantitative Objective 1: To examine predictors of citizenship (Multiple Stepwise Regression)		

Block 1 Independent variables	Block 2 Independent variables	Dependent variable
Race, ethnicity, gender, sexuality, religion	1. Everyday discrimination	1. Citizenship
	2. Discrimination with respect to gender	
	3. Discrimination with respect to sexuality	
	4. Discrimination with respect to race	
	5. Discrimination with respect to religion	
	6. Mental health stigma	
	7. Financial stress	
	8. Access to digital technology	
	9. Recovery orientation of services	

**Quantitative Objective 2: To determine if citizenship predicts recovery (Multiple Stepwise Regression)**		

**Block 1 Independent variables**	**Block 2 Independent variables**	**Dependent variable**

Significant demographic variables	1. Citizenship	2. Recovery
	2. Recovery-orientation of mental health care received	

**Quantitative Objective 3: To compare current and desired levels of citizenship-based care (Bonferroni-corrected, Pearson Correlational Analyses)**		

	**Independent variable**	**Dependent variable**

	Current perceived levels of citizenship-oriented care	Desired levels of citizenship-oriented care

### Mixed Methods Analysis

A mixed methods analysis will be conducted, whereby the findings from each component will be compared and contrasted in the discussion section using a weaving method (63). Specifically, we will produce a weaved depiction addressing how young people with mental health challenges experience citizenship; what factors shape or predict experiences of citizenship; and how one’s sense of citizenship influences recovery. This will allow us to develop an overall understanding and generate meta-inferences about experiences of citizenship among youth with mental health challenges.

## Dissemination of Findings

Findings of the study will be disseminated in peer-reviewed journals and presented at various conferences. An end-of-grant knowledge dissemination strategy will be conducted with the aim of creating social change across healthcare contexts and communities. In addition the findings from this work will be presented to all first episode psychosis programs in Quebec via AQPPEP (a Quebec-wide network aimed at dissemination knowledge and implementing best practices for treating the early phases of psychosis); with la Chaire réseau Jeunesse [Youth Network Chair], an initiative that supports systemic transformations to promote autonomy, as well as personal, social and civic development of youth in Québec; with youth mental health networks in Canada via ACCESS Open Minds, with mental health services in the United States via the New England Mental Health Technology Transfer Center, and internationally via the International Recovery and Citizenship Collaborative.

## Discussion

The overall purpose of this study is to examine citizenship among youth with mental health challenges living in Canada using mixed methods. The qualitative objectives of this study are to explore how young people’s sense of citizenship is experienced, barriers to citizenship, and how one’s sense of citizenship influences their recovery and healing. The quantitative objectives of this study are to examine rates and predictors of citizenship, to determine how citizenship affects recovery, and to examine current and desired methods of citizenship-based care. Researchers, practitioners, activists, people with lived experience, and policy makers have called for greater attention to the underlying material, social, cultural and political realities that influence recovery from mental health challenges (21). One approach to addressing these realities has been to help people build a sense of citizenship within their communities. As of yet, no study has examined citizenship among young people in Canada, who face personal, developmental, generational, and political challenges. These challenges increasingly include experiences of discrimination; risings costs of living, especially with respect to home ownership and tuition costs; and a vast urban-rural divide. Many of these have not been acknowledged by the mental health in youth mental health contexts. Our study will therefore address a substantial knowledge gap related to youth mental health.

Our study has several methodological strengths. The study will be led by a multidisciplinary team of people with varying types of expertise, different professional roles, and a broad range of personal experiences; thus, we will bring a wealth of diverse standpoints to the project. Our reliance on multiple, integrated methods to address the research objectives may offer a more nuanced and thorough understanding of the topics we will investigate. Our study is one of few to adopt an intersectional perspective in mental health research; a perspective that acknowledges how mental health doesn’t exist outside of political, institutional, social, and identity-based factors. In employing both qualitative and quantitative methods, this study may yield important insights into how this research framework can be used and applied. Overall, the findings of this research will yield important insights into the ways integrated youth mental health services in Canada may be oriented toward healing, care, and recovery; as well as the need for interventions that specifically support citizenship among users of such services.

Potential limitations of this study include that the Citizenship Outcome Measure has yet to be validated with English and French speaking youth in Canada. Some items applicable in the American context may not be applicable in the Canadian context. Many of our the other included scales have either not been validated among young people with mental health challenges or in the Canadian context. However, these are some of the most applicable scales available. Depending on sample sizes, we may be able to establish the psychometric properties of the scales, which will significantly advance knowledge on their use. Given that we are recruiting online, another limitation may include potential difficulties recruiting our targeted sample sizes for each objective. Indeed, many people may be experiencing high levels of fatigue and disinterest in engaging in online activities that may be unnecessary for their day-to-day lives. As such, we may need to broaden our recruitment strategy beyond the youth mental health services outlined in our protocol to include community organizations or other mental health services.

In addition, having to rely on online recruitment may make it difficult to recruit highly marginalized young people, such as youth experiencing homelessness or youth who don’t have access to the internet. As pandemic restrictions in Canada begin to ease, it will be a priority for our team to employ in-person recruitment from mental health services.

A future direction of this study will be to implement a citizenship-oriented intervention within integrated youth mental health services. This intervention will be community engaged and participant informed.

## Ethics Statement

This study has received Ethical Approval from the Douglas Hospital Research Centre Ethics Board. Informed consent will be obtained from all participants prior to participation.

## Author Contributions

GJ wrote the first draft of the manuscript. All authors made intellectual contributions to the manuscript, and reviewed the manuscript and provided feedback on it.

## Conflict of Interest

The authors declare that the research was conducted in the absence of any commercial or financial relationships that could be construed as a potential conflict of interest.

## Publisher’s Note

All claims expressed in this article are solely those of the authors and do not necessarily represent those of their affiliated organizations, or those of the publisher, the editors and the reviewers. Any product that may be evaluated in this article, or claim that may be made by its manufacturer, is not guaranteed or endorsed by the publisher.
